# Bioinspired magnetite synthesis *via* solid precursor phases

**DOI:** 10.1039/c6sc00523c

**Published:** 2016-06-13

**Authors:** Jos J. M. Lenders, Giulia Mirabello, Nico A. J. M. Sommerdijk

**Affiliations:** a Laboratory of Materials and Interface Chemistry , Centre for Multiscale Electron Microscopy , Department of Chemical Engineering and Chemistry , Institute for Complex Molecular Systems , Eindhoven University of Technology , PO box 513 , 5600 MB Eindhoven , The Netherlands . Email: n.sommerdijk@tue.nl

## Abstract

Living organisms often exploit solid but poorly ordered mineral phases as precursors in the biomineralization of their inorganic body parts. Generally speaking, such precursor-based approaches allow the organisms – without the need of high supersaturation levels – to accumulate significant quantities of mineral material at the desired place and time, where they can be molded and crystallized into the right morphology and structure. This strategy is also of interest in the field of bioinspired materials science, as it potentially enables the bottom-up creation of novel materials with equal or improved functionality as compared to Nature, in water and at ambient temperatures. Also for the biomineralization of magnetite (Fe_3_O_4_) such a strategy has been reported: ferrihydrite, a poorly crystalline iron oxide, has been identified as a precursor for the final magnetite phase in the magnetosomes of magnetotactic bacteria as well as in the outer layers of chiton teeth. In this perspective, we discuss the efforts of us and others to understand and tune the nucleation and growth of magnetite crystals to date, in aqueous, room-temperature syntheses and employing different solid precursor phases. The various examples demonstrate the importance of the precursor approach in controlling the different properties of magnetite nanoparticles.

## Abstract 2

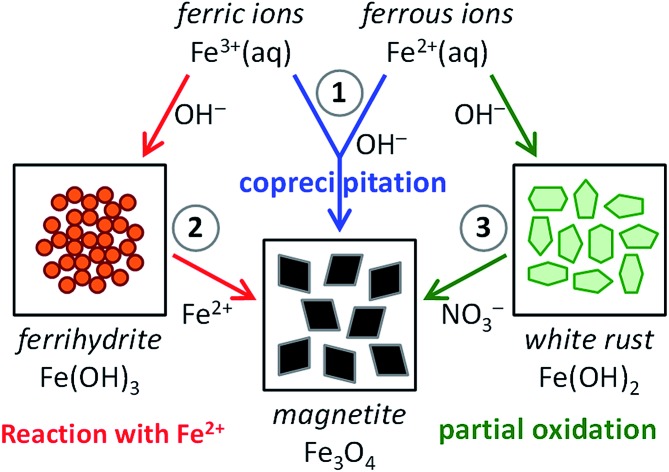
Living organisms often exploit solid but poorly ordered mineral phases as precursors in the biomineralization of their inorganic body parts.

## 1. Magnetite biomineralization

Biomineralization is the process in which organisms mineralize inorganic components to construct hybrid materials with highly specialized functions, such as bone, teeth and seashells.^1^ Through the use of biomolecular templates and additives often a high level of control over composition, structure, size and morphology of the constituent mineral components is obtained which results in materials with complex shapes and textures, exceptional structural hierarchy, and interesting properties. Magnetite (Fe_3_O_4_) is an intriguing biomineral that is used by different organisms both for its structural and magnetic properties.

Magnetite is present in the magnetoreceptive organs of migratory birds,^2^–^4^ honeybees^2^,^5^ and certain fish.^2^,^6^ It also occurs as a polycrystalline outer layer of the radular teeth of chitons,^7^,^8^ providing hardness and abrasion resistance to allow them to scrape micro-algae from rocks. Further, magnetotactic bacteria^9^ biomineralize intracellular chains of organelles, called magnetosomes,^10^–^12^ forming nanocrystals of magnetite or sometimes greigite (Fe_3_S_4_).^13^ This provides the bacteria with a microscopic internal “compass needle” with enough magnetic moment to allow them to orient along the field lines of the geomagnetic field. This processes, called magnetotaxis, is believed to aid them in finding micro-aerobic regions having the optimal oxygen concentration.^14^ However, also, alternative functions of the magnetosomes have been proposed including the sensing of oxygen concentration,^15^ storage of inorganic oxidants^16^ or proton translocation.^17^

Although several organisms are involved in magnetite formation, the biomineralization of magnetosomes in magnetotactic bacteria is the most extensively studied. Furthermore it is a beautiful example of how nature can optimize all aspects of the nucleation and growth of minerals from the nanoscale to the mesoscale, in this case to maximize the magnetic properties of the crystal chain. To start with, the crystals produced are in the 30–140 nm size range,^10^,^11^ which makes them stable single-domain ferrimagnets. The crystals consist of stoichiometric, structurally pure Fe_3_O_4_,^18^ which has a higher magnetization compared to more oxidized iron oxide.^19^ In addition, even though magnetite has a cubic unit cell and thus in principle forms crystals which are symmetrical in all three dimensions, magnetosome crystals are often encountered as rectangular or bullet-shaped.^20^,^21^ The specific morphologies are species-dependent and commonly elongated along one of the [111] magnetic easy axes of the crystal structure, which again enhances the magnetic dipole moment of the crystals.^22^ Most importantly, the magnetosomes are usually both crystallographically and magnetically aligned^23^–^25^ with the [111] axes of the crystals along cytoskeletal protein filaments,^26^,^27^ which means that they maximally contribute to the total magnetic moment of the bacterium.

To achieve such a high degree of control, the magnetite synthesis takes place within the confined space of the lipid vesicle that forms the organic outer layer of the magnetosomes.^9^,^28^ This allows crystal nucleation to be restricted to a single localized event and the subsequent growth of a crystal with defined dimensions. Moreover, the entire biomineralization process is under strict control of a specialized set of proteins,^11^,^29^,^30^ directing all stages of the magnetosome formation, from the vesicle formation by membrane invagination to the iron uptake,^31^ nucleation, growth and assembly of the magnetite crystals.^32^–^34^ To synthesize magnetite the bacteria can take up both Fe^3+^ and Fe^2+^ from their environment, while the oxygen in the magnetite is known to originate from water^35^ with the crystal formation most probably occurring at basic pH and under mildly reducing conditions.^36^,^37^ The specific iron chemistry involved has been a topic of prolonged debate,^37^–^39^ but now there is growing evidence for the presence of a ferrihydrite-like intermediate inside the magnetosome vesicle prior to the formation of magnetite.^40^,^41^ This intermediate was proposed to form from a highly disordered phosphate-rich ferric oxide phase.^41^ While in the case of the radular teeth of chitons the magnetite formation was shown to occur through a partial reduction of ferrihydrite,^42^–^46^ for magnetosome formation this part of the process is not yet resolved, but seems most likely to proceed by the addition of Fe^2+^ to the ferrihydrite precursor.^47^

The crystallization from precursors avoids conditions of high supersaturation and toxic iron levels inside the cell, and is thought – together with the compartmentalization – to prohibit uncontrolled nucleation followed by limited growth. This precursor based approach is certainly not limited to magnetite only, but in fact is widely spread amongst the crystalline biominerals, with amorphous calcium carbonate (ACC) as a precursor for calcite^48^ or aragonite^49^ and amorphous calcium phosphate (ACP) as a precursor for apatite^50^ being the most well-known.

## 2. Bioinspired magnetite synthesis

### 2.1 General aspects

Inspired by the beautiful examples that nature offers, materials scientists are aiming to capture the key aspects of precursor-based biomineralization processes in biomimetic crystallization experiments to obtain control over the nucleation and growth of minerals.^1^ For common biominerals, such as silica,^51^,^52^ calcium carbonate^53^,^54^ and calcium phosphate,^55^,^56^ many activities have focused on their biomimetic synthesis, employing solid precursor phases and (macro)molecular additives (amino acids, peptides and proteins, surfactants and block copolymers, polyelectrolytes), interfaces (Langmuir or self-assembled monolayers) and/or templates (gels, porous membranes, colloidal crystals of latex particles) to create materials with controlled morphology and structure. Compared to these huge bodies of work, the amount of research dedicated to the bioinspired crystallization of magnetite is still relatively small.^57^–^59^ Nonetheless, mimicking the pathways to magnetite in biomineralization processes as encountered in magnetotactic bacteria and chitons will aid not only in understanding the generic principles of biomineralization, but also in finding routes to the aqueous, room-temperature production of magnetite nanoparticles, with control over their dimensions and organization and thereby their magnetic properties.

**Fig. 1:**
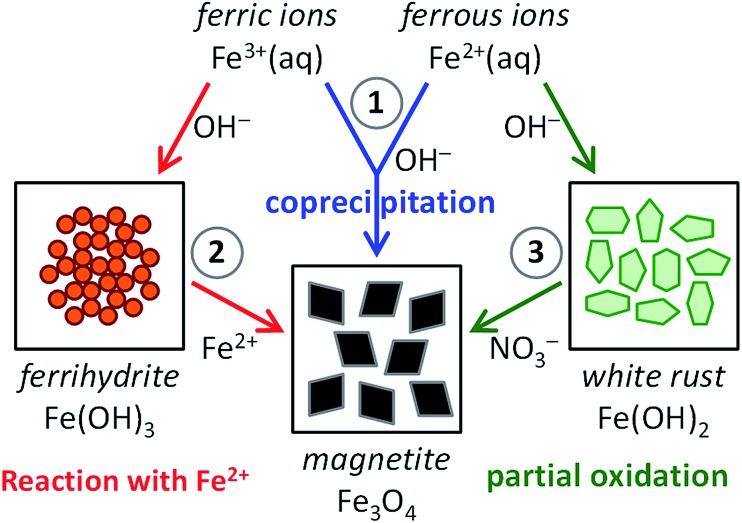
Scheme visualizing the three main synthesis routes to magnetite (Fe_3_O_4_): (1) magnetite formation by controlled coprecipitation from both Fe^3+^ and Fe^2+^ ions, (2) magnetite formation from Fe^3+^ ions through a solid ferrihydrite (Fe(OH)_3_) precursor and Fe^2+^ ions by ammonia diffusion, and (3) magnetite formation from Fe^2+^ ions through a solid white rust (Fe(OH)_2_) precursor by partial oxidation with NO_3_^–^.

In this perspective, we discuss all recent activities aiming at this goal. For this we organized the current scientific literature in three main categories, based on the synthesis route chosen (see Fig. 1): (1) magnetite formation by controlled coprecipitation of Fe^3+^ and Fe^2+^ ions, (2) magnetite formation from reaction of Fe^2+^ ions with a solid ferrihydrite precursor (Fe^III^), and (3) magnetite formation from the partial oxidation of a solid white rust precursor (Fe^II^) with NO_3_^–^. Note that routes (1) and (2) can both be referred to as coprecipitation reactions in the general sense of the term. Moreover, ferric and ferrous ions lead to the formation of different precursor phases (see Section 2.3 and 2.4) due to their different solubility.

From the examples discussed here, it will become clear that controlling the crystallization kinetics through a slow supply of the reactants – by means of titration, diffusion or the conversion of solid precursor phases – is an important step in controlling the properties of magnetite nanocrystals, and only under such controlled conditions organic additives can be employed to further direct nucleation and/or growth.

### 2.2 Magnetite synthesis by coprecipitation of Fe^2+^ and Fe^3+^

The most straightforward method to obtain magnetite synthetically is the coprecipitation of Fe^3+^ and Fe^2+^ in alkaline conditions according to eqn (1) (route (1) in Fig. 1), which simply can be carried out in water and at room temperature under an inert atmosphere. This method was described for the first time already in 1852 by Lefort^60^ and it was popularized by Kiyama^61^ and Massart^62^,^63^ in the seventies and eighties of the last century.

1 2Fe^3+^ + Fe^2+^ + 8OH^–^ → Fe_3_O_4_ + 4H_2_O

However, as magnetite is only sparingly soluble in basic media, having an equilibrium iron concentration of ∼0.02 μM at pH 10 and 25 °C,^64^ and the introduction of the acidic Fe^3+^/Fe^2+^ mixture into a highly alkaline solution leads to instant magnetite precipitation. This typically results in small nanoparticles with diameters < 20 nm that due to the limited size of the magnetic domain have superparamagnetic properties. Unfortunately the synthesis procedure provides little means of control over the size (distribution) and morphology,^65^,^66^ although the iron concentration and aging time^67^ as well as the type of counter anion^68^ have been found to affect the average particle size. Also, these nanoparticles can be coated with organic (macro)molecules to enhance their water dispersibility.^69^–^73^

Coprecipitation can also be performed by adding the base to the mixed valence iron ion solution to precipitate the magnetite nanoparticles as the pH rises. This reaction sequence was used in a biomimetic context, by increasing the pH of a mixture containing Fe^3+^/Fe^2+^ and Mms additives.^74^,^76^,^77^ In these experiments the magnetosome proteins Mms6 (ref. 34), MamC^74^ and MmsF^75^ in isolated form were employed as additives aiming at regulating the formation of magnetite nanoparticles. This typically allowed for the formation of better defined crystals as compared to the control experiments. However, additional measures such as a gel medium^77^ or a carbonate buffer^74^ are often used to slow down the precipitation kinetics, thereby enabling protein additives to impact magnetite nucleation and growth. Room-temperature coprecipitation of Fe^3+^ and Fe^2+^ inside a gelatin medium was also used to create thermoreversible magnetic hydrogels with varying degrees of crosslinking and particle loadings.^78^ Furthermore, decreasing the base addition rate allows time to probe the formation mechanism and the effect of additives on it,^79^ as well as for the detection of precursor phases (see next section).

### 2.3 The role of ferrihydrite in magnetite coprecipitation

**Fig. 2:**
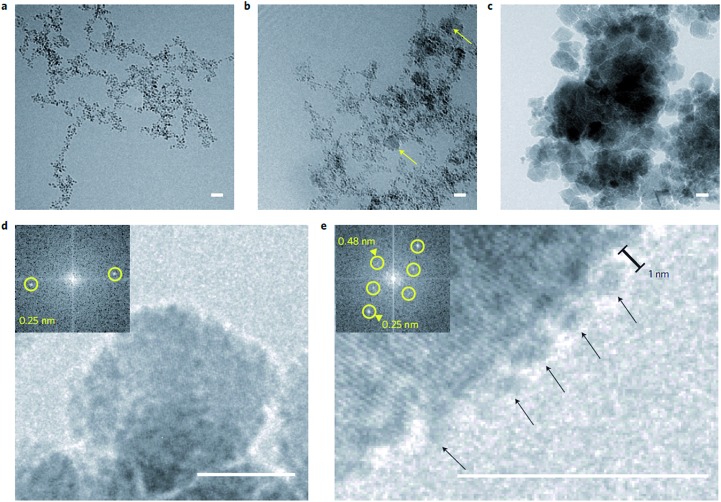
Magnetite formation from a ferrihydrite-like precursor phase by controlled coprecipitation. (a–c) Cryo-TEM time series of the evolving primary-particle and magnetite-nanoparticle aggregates as imaged after (a) 2 min, (b) 6 min and (c) 82 min. Yellow arrows in (b) indicate early formed crystalline magnetite nanoparticles. (d) Image of a magnetite nanoparticle. (e) Image of primary particles (arrows) attaching to the surface of a magnetite nanoparticle. Insets in (d and e): fast Fourier transform indicating the crystallinity of the particles. Scale bars: 10 nm. Reproduced from ref. 80.

Baumgartner *et al.* reported an interesting method to obtain control over the reaction kinetics of the coprecipitation method, employing slow but continuous titration of 2Fe^3+^:Fe^2+^ mixtures while keeping the reaction pH constant.^80^–^82^ This process allowed the controlled formation of magnetite through the conversion of an *in situ* generated nanoparticulate, ferrihydrite-like precursor phase (Fig. 2).^80^ Further, it was demonstrated that growth proceeded by attachment of the precursor particles to the crystal surfaces, followed by dehydration. Although the controlled dosing of reactants indeed allowed for continuous crystal growth up to sizes of about 40–50 nm and thus well beyond the superparamagnetic regime (∼20 nm), it could not fully suppress ongoing nucleation of new particles, leading to rather polydisperse products.^81^ Nevertheless, when poly(l-arginine) was used as a positively charged crystallization control agent, the resulting crystals became colloidally stabilized in dispersion and their size distribution was significantly reduced.^82^ This effect is due to the interaction between positively charged additives and the negatively charged surface of magnetite crystals, which lowers the energy surface and promotes nucleation. In contrast, under the employed reaction conditions, negatively charged macromolecules (poly(l-glutamic acid) and the MamJ and MtxA_Δ1–24_ proteins) were found to completely inhibit magnetite formation. This effect is due to the interaction between negatively charged additives and iron ions, which stabilize and induce the formation of an amorphous precursor state.

**Fig. 3:**
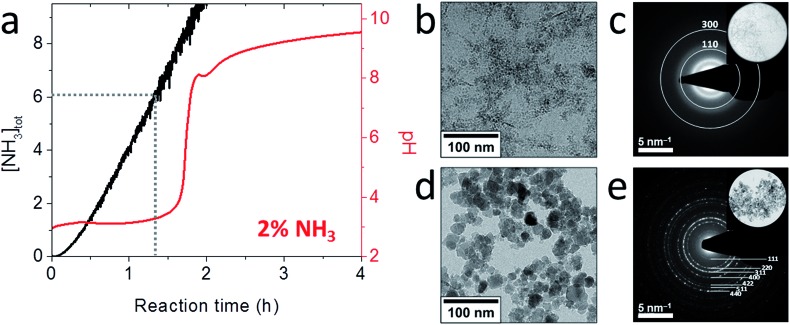
Magnetite formation from a ferrihydrite precursor phase by ammonia diffusion. (a) Evolution of pH and total NH_3_ concentration over time during 2 vol% NH_3_ diffusion in 30 mL of 3 mM Fe solution. (b) Cryo-TEM image after 1 h reaction time that shows nanoparticulate ferrihydrite. (c) Low-dose SAED pattern of the precursor indexed to 2-line ferrihydrite. Inset: selected area. (d) TEM image of the resulting 17 ± 8 nm magnetite nanoparticles. (e) SAED pattern of the nanoparticles indexed to magnetite. Inset: selected area. Reproduced from ref. 83.

To more closely mimic the iron chemistry in magnetite biomineralization, which involves the formation of the ferrihydrite precursor phase as a distinct first step (route (2) in Fig. 1), we developed an ammonia (NH_3_) diffusion method^83^ in analogy with the ammonium carbonate ((NH_4_)_2_CO_3_) diffusion method for bioinspired calcium carbonate (CaCO_3_) crystallization.^53^,^84^ In this approach, we use a closed system containing an inert atmosphere in which we let NH_3_ evaporate from an aqueous solution and diffuse into a 2Fe^3+^:Fe^2+^ solution that is being stirred. The influx of NH_3_ will increase the pH of the iron solution, reducing the solubility of Fe^3+^ and Fe^2+^ and forcing them to precipitate in different stages of the process.^85^ After an initial increase the pH of the iron solution stabilizes at ∼3 (Fig. 3a) where the incoming base is consumed by the reaction with Fe^3+^ forming 2-line ferrihydrite (Fe(OH)_3_, Fig. 3b and c) according to eqn (2) and (3):

2 NH_3_ + H_2_O → NH_4_^+^ + OH^–^

3 Fe^3+^ + 3OH^–^ → Fe(OH)_3_

After sufficient NH_3_ has diffused in to precipitate nearly all Fe^3+^, the pH increases rapidly up to ∼7.8 (Fig. 3a), where a local maximum in the pH curve marks the formation of magnetite (Fe_3_O_4_, Fig. 3d and e) by reaction of the ferrihydrite precursor with the Fe^2+^ still present in solution according to eqn (4):

4 2Fe(OH)_3_ + Fe^2+^ + 2OH^–^ → Fe_3_O_4_ + 4H_2_O

**Fig. 4:**
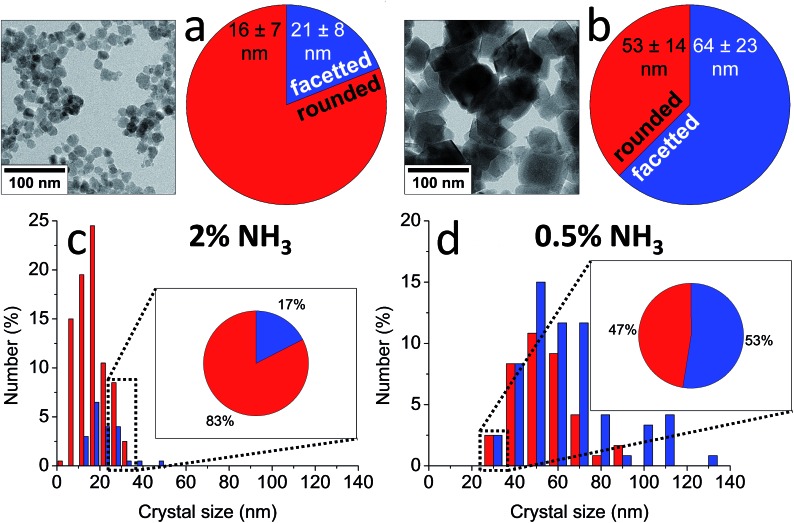
(a and b) TEM images and shape distributions and (c and d) size distributions of magnetite nanoparticles obtained by (a and c) 2 vol% NH_3_ and (b and d) 0.5 vol% NH_3_ diffusion in 30 mL of 3 mM Fe solution, which show a trend from smaller, rounded particles to larger, facetted crystals. Insets in (c and d): shape distributions of the 25–35 nm size fractions in each sample, which show a similar trend. Reproduced from ref. 83.

Indeed, the kinetics of the process are determined by the balance between the starting amount of iron and the NH_3_ influx, and thus can be directed at will by changing the concentrations of the iron and the NH_3_ solutions and/or the reaction volume. Both reactant concentrations directly affect the nucleation density, meaning that the average dimensions of the resulting magnetite crystals can be controlled as well. The particle sizes spanned from 15 ± 4 nm to not less than 60 ± 21 nm for the highest and lowest iron and NH_3_ concentrations, respectively (Fig. 4c and d). The morphology and magnetic properties of the products were concomitantly varied between superparamagnetic, mostly rounded particles to single-crystalline octahedra showing stable-domain ferrimagnetic behavior (Fig. 4a and b). The faceted morphology of the latter crystals at first sight seemed coupled to their larger size, while the rounded morphologies were associated with smaller diameters. However, comparing fractions of crystals with equal size from different samples (insets in Fig. 4c and d), showed that in fact the development of facets was related to the longer growth period of the crystals and not to the crystal size, stressing the importance of controlling the crystallization kinetics.

**Fig. 5:**
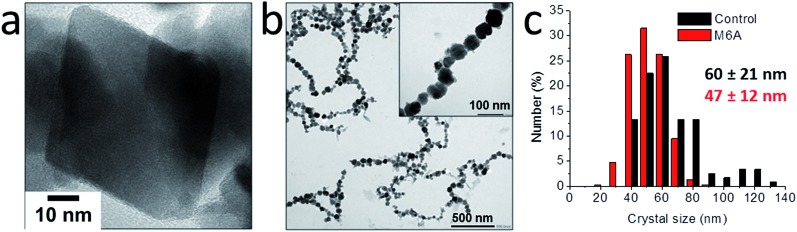
Characterization of the magnetite crystals obtained by 0.5 vol% NH_3_ diffusion in 30 mL of 3 mM Fe solution in the (a and c) absence and (b and c) presence of 0.3 mg mL^–1^ M6A. (a) HRTEM image of a representative octahedral crystal. (b) TEM image of the rounded crystals aligned in strings. Inset: higher magnification. (c) Size distributions of the crystals obtained with and without M6A. Reproduced from ref. 83.

Although the ammonia diffusion method allowed the synthesis of magnetite with a rather wide range of average particle sizes from a solid ferrihydrite precursor phase, no real control over the polydispersity of the products was obtained. However, the developed precursor-based approach allowed soluble macromolecular additives to further direct the crystal size (distribution) and shape,^86^ for example by employing the M6A peptide – the active C-terminal part of the Mms6 protein^87^ (Fig. 5).^83^ The use of M6A did not only result in a reduction of the particle size distribution, it also changed the morphology of the particles from facetted to rounded, demonstrating the action of M6A in influencing the growth of magnetite in addition to synchronizing the nucleation. Similar as for poly(l-arginine),^82^ the interaction between M6A and the crystals also allowed their colloidal stabilization in aqueous dispersion and alignment in long strings, due to the attractive ferrimagnetic forces between them as a result of their stable-domain character. These effects were attributed to the negatively charged aspartic acid (D) and glutamic acid (E) moieties in M6A, as a control peptide in which those residues were replaced did not have any impact on the crystallization process.

Similarly, to further investigate the effect of the different types of amino acids on the nucleation and growth of magnetite, we designed and synthesized two libraries of copolypeptides, with varying amino acid composition. These libraries are used to study the effects of monomer composition, and physicochemical properties such as net charge and polarity on magnetite crystallization. The first library comprised aspartic acid (D) and serine (S)^88^ and the other one comprised glutamic acid (E), lysine (K) and alanine (A)^89^ as the monomers. These copolymers were produced with equal length (degree of polymerization = 24) and precise amino acid composition, but with random monomer sequence and therefore hardly any secondary structure^89^ such that the observed effects could be assigned to the amino acid composition rather than to the presence or absence of secondary and tertiary structures.

When these polypeptides were use as additives in this ferrihydrite based route to magnetite,^86^ we found that increasing either the relative copolypeptide concentration (*i.e.* the amino acid/iron ion ratio) or the acidic amino acid (*i.e.*, aspartic acid or glutamic acid) content of the polypeptides led to a gradual decrease of the obtained particle dimensions from 60 ± 21 nm down to 11 ± 6 nm. This reduction in size went hand in hand with decreasing saturation/remanent magnetization values and coercivities (down to complete superparamagnetic behavior), and a more and more rounded morphology. In contrast, varying the amount of lysine residues – which are positively charged at the pH values used – in the polymers had no observable effect on the size or shape of the magnetite particles.^86^ However, Rawlings *et al.* have showed how the use of proteins rich in lysine can direct the formation of magnetite nanocubes in aqueous room temperature reaction, due to the interaction with [100] crystal face.^90^

It was shown that the more negatively charged polypeptides, through the interaction of their negatively charged residues with the Fe^2+^ ions in solution (the Fe^3+^ had already precipitated as FeH), delayed nucleation, pushing the nucleation point to higher pH values. Consequently nucleation occurred at later time points where supersaturation had built up to higher values, where the higher nucleation rates lead to smaller crystal sizes.

**Fig. 6:**
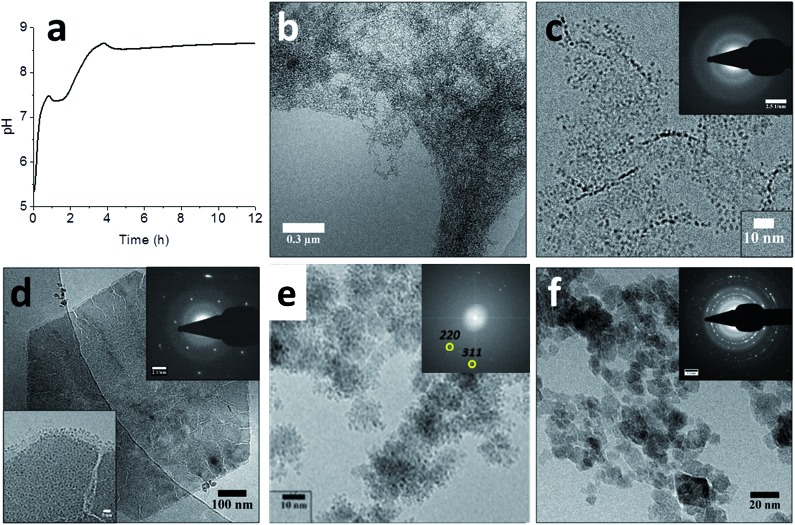
Magnetite formation from a ferrihydrite precursor phase by ammonia diffusion. (a) pH curve through time for magnetite synthesis from ferrihydrite and Fe^2+^ upon in-diffusion of NH_3_, showing two distinct nucleation events. (b–f) Cryo-TEM images of (b) the ferrihydrite precursor, showing its gel-like character, (c) ferrihydrite stabilized by Fe^2+^ at pH ∼ 5, showing the 1.5–2.0 nm primary particles (inset: SAED pattern, showing no crystallinity), (d) the hexagonal green rust intermediate after ∼1 hour reaction time (insets: SAED pattern with 3.9 nm^–1^ spacings and higher magnification of a corner of the crystal, showing the 1.0–1.5 nm secondary particles), (e) the formation of magnetite through the aggregation of secondary particles (inset: FFT pattern showing lattice spacings of magnetite, while the contours of the secondary particles are still clearly visible in the aggregates), and (f) the final magnetite product (inset: SAED pattern, displaying the common magnetite reflections). Please note that the cryoTEM image in (e) was recorded in a solution with higher Fe^2+^ concentration than the other images. Reproduced from ref. 91.

To studied the mechanism of this reaction in more detailed the ammonia diffusion method was modified: the Fe^2+^ was now added in a separate, second step to an earlier formed 6-line ferrihydrite precursor instead of being added simultaneously with the Fe^3+^ (Fig. 6).^91^ A detailed cryoTEM analysis showed that the 6-line ferrihydrite precursor material was present as a hydrated gel-like nanoparticulate network (Fig. 6b) that upon addition of the Fe^2+^ at pH ∼5 dehydrated to form well-defined 1.5–2.0 nm primary ferrihydrite–Fe^2+^ particles (Fig. 6c).^80^ The FeH–Fe^2+^ secondary particles were subjected to the in-diffusion of ammonia, after which two subsequent nucleation events indicated by the uptake of base could be identified at pH ∼ 7.5 and pH ∼ 8.7 (Fig. 6a).

The first nucleation event indicated by the pH curve represented the formation of 200–500 nm hexagonal platelets of green rust, an Fe^2+^-rich iron oxide phase (Fig. 6d). High-resolution imaging showed that these platelets had cracks and that their surfaces and edges were covered with 1.0–1.5 nm particles (Fig. 6d, inset), which were smaller than the 1.5–2.0 nm primary particles. The concomitant observations of cracks and the 1.0–1.5 nm secondary particles suggested that the latter formed through the re-dissolution of the green rust platelets acting as a source of Fe^2+^. The second event which occurred after the pH has risen to ∼8.7 marked the formation of magnetite crystals from the secondary particles on the surfaces of the platelets (Fig. 6e), which after 16 hours became the single product at the expense of both the green rust and the nanoparticles (Fig. 6f). Hence, it appears that this aggregation-based crystallization process is key to the continuous growth of magnetite with control over the size and shape of the resulting nanocrystals.

### 2.4 Magnetite synthesis involving white rust precursors

Another aqueous route to magnetite through a different solid precursor phase is the partial oxidation of Fe^2+^ (route (3) in Fig. 1), which is also performed in alkaline conditions. Although earlier records exist,^92^,^93^ this method was first extensively discussed by Sugimoto and Matijević in 1980,^94^ and has received increasing attention since then.^95^ In this approach, Fe^2+^ is initially precipitated at high pH as ferrous hydroxide according to eqn (5) (Fe(OH)_2_, white rust), and subsequently oxidized and recrystallized to magnetite according to eqn (6), usually by means of potassium nitrate (KNO_3_).

5 Fe^2+^ + 2OH^–^ → Fe(OH)_2_

6 3Fe(OH)_2_ + NO_3_^–^ → Fe_3_O_4_ + NO_2_^–^ + 3H_2_O

As opposed to coprecipitation, in partial oxidation the reaction kinetics are determined by the Fe^2+^ oxidation rate, which increases at increasing pH.^94^–^96^ This makes the partial oxidation method highly dependent on the (relative) iron, base and oxidant concentrations.^59^,^97^–^100^ Under optimized conditions the method produces phase-pure magnetite crystals with sizes in the stable single-domain. In most cases, the reaction is carried out at elevated temperatures (typically 90 °C), although it has been demonstrated that complete conversion to magnetite can also be achieved at ambient temperatures.^100^–^102^

Also in partial oxidation reactions, the addition of the Mms6 protein^76^ and the M6A peptide^87^ (*vide supra*) have been explored to obtain biomimetic size and shape control over magnetite formation. Indeed both biomacromolecules were able to modify the magnetite morphology from octahedral to cubo-octahedral through stabilization of the ) have been explored to obtain biomimetic size and shape control over magnetite formation. Indeed both biomacromolecules were able to modify the magnetite morphology from octahedral to cubo-octahedral through stabilization of the 〈100} facets, similar as what is observed for bacterial magnetite. Arakaki 100} facets, similar as what is observed for bacterial magnetite. Arakaki *et al.* suggested that the acidic residues of Mms6 are responsible for the stabilization of the [100] face.^87^ However, this stabilization has also been observed through interaction with basic residues.^90^

Further, the attachment of Mms6 to specific areas of self-assembled monolayers was used to induce the selective nucleation and growth of magnetite nanoparticles through partial oxidation of the Fe(OH)_2_ precursor in those areas, thereby creating surfaces patterned with arrays of immobilized magnetite crystals.^103^ Together these studies showed that also in the partial oxidation method Mms6 was able to control both the nucleation and growth of magnetite from a solid precursor.^76^,^103^

**Fig. 7:**
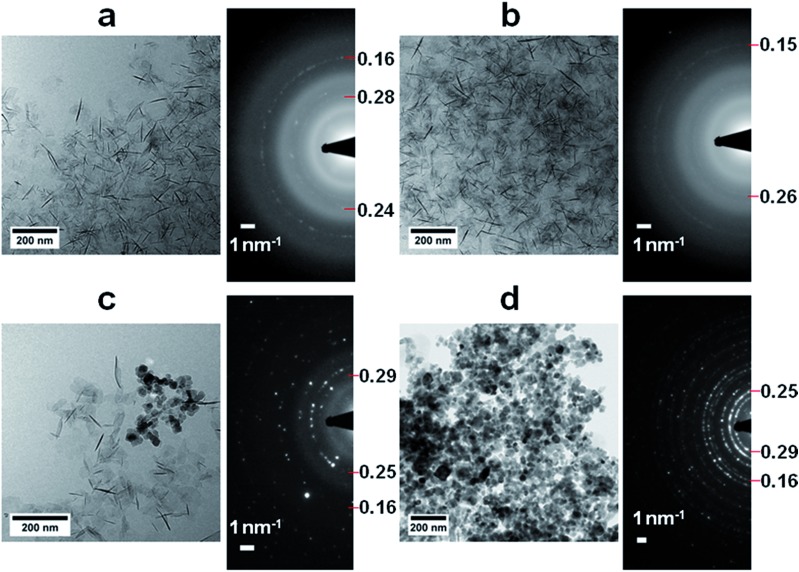
Magnetite formation from a white rust precursor phase by partial oxidation. (a–d) Cryo-TEM images and corresponding SAED patterns of (a) the product after addition of Fe^2+^ to the base solution, showing the formation of the Fe(OH)_2_ precursor phase, (b) the product 1 h after the addition of nitrate, showing additional reflections (indicated) but no morphological changes, (c) the product 2 h after the addition of nitrate, showing partial conversion to magnetite crystals, and (d) the product 24 h after the addition of nitrate, showing diffraction rings typical for magnetite. Reproduced from ref. 102.

A detailed cryoTEM study into the role of the precursor showed that the reaction started with the precipitation of ∼100 nm hexagonal white rust platelets (Fig. 7a) which subsequently transformed into more oxidized green rusts after 1 hour with conservation of the morphology (Fig. 7b), before redissolving and reprecipitating as magnetite crystals (2 hours and later, Fig. 7c and d).^102^ Time-resolved cryo-TEM indicated that this reaction gave well-crystallized 34 ± 11 nm particles, which for 65% had an octahedral morphology according to cryo-TEM and cryo-electron tomography (cryo-ET, 3D cryo-TEM). Also in this study the slow recrystallization kinetics from a solid precursor phase to magnetite allowed polymeric additives to tweak the properties of the obtained nanoparticles. We could demonstrate that poly((α,β)-d,l-aspartic acid) (pAsp) can be employed as a negatively charged nucleation and growth control agent in this reaction, allowing the formation of smaller but better-defined nanoparticles, which for 85% had a fully rounded morphology.

## 3. Conclusions and outlook

In conclusion, approaches employing solid precursor phases so far have enabled synthesis routes that, in water and at room temperature, allow tuning of the phase purity, size (distribution), morphology, magnetic properties, dispersibility and organization of magnetite nanoparticles, also beyond the superparamagnetic regime. Through control of the reaction kinetics, the average crystal size can be adjusted from ∼10 to ∼60 nm, enabling magnetic properties ranging from superparamagnetic to stable single-domain ferrimagnetic behavior. While there is still room for improvement in the control over crystal size distribution and morphology, the methodologies that have been developed so far have provided significant steps to achieve these goals. Further, the precursor phase concept likely can be translated to the bioinspired synthesis of other functional materials.^57^

Although the experimental control over the average size of magnetite nanocrystals was achieved, none of the designed bio-inspired strategies so far resulted in truly monodisperse particles, which would be an asset for their use in many technological applications. In bulk aqueous synthesis such a situation can only be reached by creating conditions that allow a well-defined nucleation event in a narrow time window, followed by an extended growth period. In this way, the particles would all start and stop growing at the same time, thereby obtaining the same size. In practice, however, conditions of high supersaturation result in instant nucleation but limited growth, because the reactants are rapidly consumed, while conditions of lower supersaturation are found to enable longer growth periods but generally do not limit nucleation events to the initial stages.

In biomineralization, minerals are often formed in confined space. For instance, apatite in bone forms inside collagen fibers, aragonite tablets in nacre grow within extracellular compartments defined by layers of chitin, while the magnetite in magnetotactic bacteria forms inside magnetosome vesicles. These organic matrices provide physical constraints to the growing crystals, thereby setting boundaries to the dimensions they can obtain. In this way, minerals with controlled sizes are created without the need to synchronize their individual nucleation and growth stages.

In further research, this concept could be implemented in aqueous magnetite crystallization by utilizing templates, such as protein cages, vesicles or porous membranes, to obtain controlled dimensions by limiting the growth of the final crystals instead of by synchronizing their nucleation. In fact this concept was first already used in the early 90's by Meldrum *et al.* who synthesized superparamagnetic magnetite nanoparticles of ∼6 nm in size using the protein cage apo-ferritin as a template.^104^ In such experiments, precursor phases may be employed to bring sufficient quantities of mineral to the desired location without need for high reactant concentrations. Nevertheless, to achieve single domain magnetite crystals, nucleation inside these template should be limited to a single event – as was shown for example for the formation of rod-like single crystals of calcium carbonate^105^ – which will be more difficult for larger templates.

Hence, while this perspective presents current methodologies to direct magnetite formation in water and at room temperature, they should be extended with additional strategies, such as crystal formation in confinement, to bring the degree of control over magnetite nucleation and growth another step further.^106^ Further, the combination of such approaches may ultimately lead to the ‘green’ synthesis of truly monodisperse magnetite crystals.
